# The Golden Approach of Trauma. Welche Blutprodukte werden zur Optimierung der präklinischen Traumaversorgung benötigt?

**DOI:** 10.1007/s00101-024-01482-6

**Published:** 2024-11-18

**Authors:** Maximilian Lothar Bamberg, Christian Grasshoff, Jessica Gerstner, Matthias Fabian Boos, Michael Bentele, Tim Viergutz, Johann Fontana, Peter Rosenberger, Robert Wunderlich

**Affiliations:** 1 Abteilung für Anästhesiologie und Intensivmedizin, BG Unfallklinik Tübingen, Tübingen, Deutschland; 2 Universitätsklinik für Anästhesiologie und Intensivmedizin, Universitätsklinikum Tübingen, Hoppe-Seyler-Str. 3, 72076 Tübingen, Deutschland; 3 Ausbildungszentrum für Notfallmedizin (NOTIS e. V.), Engen, Deutschland

**Keywords:** Notfallmedizin, Trauma, Prähospital, Transfusion, Gerinnungsprodukte, Emergency medicine, Trauma, Prehospital care, Transfusion, Coagulation

## Abstract

**Hintergrund:**

Die „Golden Hour of Trauma“ bezeichnet die kritische erste Stunde nach einem schweren Trauma, in der eine rechtzeitige medizinische Versorgung entscheidend ist. Diese Studie fokussiert sich auf die Optimierung der Traumaversorgung durch an die jeweilige Verletzung angepasste Behandlungen statt nur auf die Geschwindigkeit der Versorgung. Ziel war es, den Verbesserungsbedarf der präklinischen Traumaversorgung, insbesondere durch den Einsatz von Blut- und Gerinnungsprodukten, zu erfassen.

**Methoden:**

Ein Online-Fragebogen wurde nach Pilotierung an Ärztinnen und Ärzte sowie Rettungsdienstpersonal gesendet, um deren Einschätzungen zur Traumaversorgung und speziell zur Nutzung von Blutprodukten und Gerinnungspräparaten zu erheben. Die Bewertung von 9 spezifischen Blut- und Gerinnungsprodukten erfolgte mittels einer 5‑stufigen Likert-Skala.

**Ergebnisse:**

Von 9837 erreichten Personen beantworteten 371 den Fragebogen, wobei Daten von 252 Personen in die Analyse einflossen. Die Mehrheit der Ärztinnen und Ärzte (89,1 %) sowie des Rettungsdienstpersonals (90,8 %) sah die Notwendigkeit, die präklinische Traumaversorgung durch den Einsatz von Blutprodukten und Gerinnungspräparaten zu verbessern. Besonders befürwortet wurden Erythrozytenkonzentrat (76,2 %) und Fibrinogen (67,1 %) zur Verbesserung der Überlebenschancen bei erheblichem Blutverlust.

**Diskussion:**

Die Ergebnisse zeigen eine Bereitschaft zur Änderung der Traumaversorgung und bestätigen effiziente Möglichkeiten hierzu. Der Schwerpunkt verlagert sich von der isolierten Zeitkomponente hin zur Qualität der Versorgung in einem optimierten Zeitintervall, was zu einem „Golden Approach of Trauma“ führen könnte.

**Zusatzmaterial online:**

Die Online-Version dieses Artikels (10.1007/s00101-024-01482-6) enthält den zugrunde liegenden Fragebogen. Bitte scannen Sie den QR-Code.

## Abstract (en)

**Background:**

The golden hour of trauma denotes the critical first hour after severe injury where timely medical response is crucial, although scientific support for this time frame is inconsistent. This study emphasizes optimizing trauma care by tailoring treatment to the specific injury rather than focusing solely on the speed of treatment. The aim is to document the need for improvement in prehospital trauma care, particularly by the use of blood and coagulation products.

**Methods:**

After a pilot study, a purpose-designed online questionnaire targeted at German physicians and rescue service personnel was utilized to collect their views on general trauma care and specifically on the use of blood and coagulation products in prehospital settings. It also assessed the appropriateness of nine specific blood and coagulation products via a 5-point Likert scale. The percentages for each item were calculated for both physicians (*n* = 110) and rescue service personnel (*n* = 142) separately as well as an overall score to delineate patterns of agreement or disagreement.

**Results:**

The study reached 9837 individuals, whereby 371 initially answered the questionnaire and 252 participants from Germany were finally included in the statistical analysis. The majority of both physicians (89.1%) and rescue service personnel (90.8%) agreed on the need to improve prehospital trauma care, particularly through the use of blood and coagulation products. Specifically, 60.9% of physicians and 83.8% of rescue personnel supported the prehospital administration of these products. Red blood cell concentrates and fibrinogen were notably endorsed, with 76.2% and 67.1% approval, respectively, for their potential to enhance survival in patients with significant blood loss; however, opinions varied on other blood products.

**Conclusion:**

The data demonstrated a readiness to change the trauma approach and confirmed that effective options are available. The utilization of certain products is supported by existing research, underlining the need for their practical implementation in preclinical settings. Here, the emphasis shifts from the isolated time components to the quality of care delivered in an optimized time interval. Ideally, timely and high-quality care should complement each other, leveraging all available therapeutic resources. This could lead to the development of a golden approach to trauma to optimize outcomes in trauma care.

## Hintergrund

„There is a golden hour between life and death. If you are critically injured, you have less than 60 min to survive. You might not die right then; it may be three days or two weeks later—but something has happened in your body that is irreparable.“ (R. A. Cowley, 1917—1991) [1] – Geprägt von diesem Gedanken entstand das Konzept der präklinischen Versorgung in der „Golden Hour of Trauma“. Ziel ist hierbei, Patientinnen und Patienten innerhalb einer Stunde nach Trauma eine definitive medizinische Versorgung zukommen zu lassen. Zunächst steht die vorübergehende Versorgung stärkster, lebensbedrohlicher Blutungen im Vordergrund. Hierbei dient das vorangestellte „c/x“ des ABCDE(„airway“, „breathing“, „circulation“, „disability“, „exposure/environment“)-Algorithmus als Handlungsleitfaden. Zuerst im Militär etabliert mittels <c>ABC („catastrophic haemorrhage“) [2], dann auch im zivilen Rettungsdienst durch cABCDE („critical haemorrhage“) oder xABCDE („exanguinating haemorrhage“) umgesetzt [3, 4]. Gelingt dies nicht, so ist nach diesem Prinzip mit einer erhöhten Morbidität und Mortalität zu rechnen. Aus dieser Grundüberlegung heraus entstanden zwei unterschiedliche notfallmedizinische Versorgungsansätze: das Paramedic-basierte „Scoop-and-Run“- und das notarztbasierte „Stay-and-Play“-Prinzip [5]. Eine Weiterentwicklung ist „Treat and Run“, das neben der medizinischen Versorgung einen schnellen Transport vorsieht, um die „Golden Hour of Trauma“ einzuhalten. Diesem Ansatz mangelt es jedoch an wissenschaftlichen Beweisen, da kein Zusammenhang zwischen der Transportzeit und dem Überleben oder dem Patienten-Outcome nachgewiesen werden konnte [6–9].

Angesichts der notärztlichen Kompetenz kann ein notarztgestütztes System kritische therapeutische Eingriffe unmittelbar an der Einsatzstelle durchführen und damit die Überlebenswahrscheinlichkeit deutlich erhöhen. Dieser Fähigkeit sollte man sich bedienen. In diesem Zusammenhang geht es nicht nur um Versorgungszeit, sondern auch um die Anpassung der Behandlung an das Verletzungsmuster und die damit verbundenen pathophysiologischen Prozesse. Beim präklinischen hämorrhagischen Schock gibt es noch Verbesserungspotenzial. In diesem Zusammenhang ist die präklinische Transfusion verschiedener Blut- und Gerinnungsprodukte zur Optimierung der Sauerstoffversorgung und der Gerinnung eine noch zu wenig genutzte therapeutische Option [10, 11].

Es stellt sich die Frage, ob am Paradigma der „Golden Hour of Trauma“ und damit am Zeitaspekt festgehalten oder auf ein qualitätsorientiertes Konzept umgeschwenkt werden sollte. Dabei geht die umfassende Nutzung aller notwendigen therapeutischen Möglichkeiten zur Optimierung der Überlebenswahrscheinlichkeit von Patientinnen und Patienten idealerweise Hand in Hand mit schnellen Versorgungs- und Transportzeiten.

Ziel dieser Querschnittstudie war, die Ansichten zweier Berufsgruppen in der Notfallmedizin – Rettungsdienstpersonal sowie Ärztinnen und Ärzte – zur Notwendigkeit der Verbesserung der präklinischen Traumaversorgung durch die frühzeitige Gabe von Blut- und Gerinnungsprodukten zu untersuchen.

## Methoden

### Studiendesign

Die Datenerhebung erfolgte über einen Zeitraum von 3 Monaten zwischen Oktober 2023 und Januar 2024. Eine anonyme, webbasierte Umfrage für Ärzte und Rettungsdienstpersonal wurde nach Pilotierung 2‑malig über die Social-Media-Kanäle der ausführenden Zentren sowie der Deutschen Gesellschaft für Rettungswissenschaften verbreitet.

### Datenerhebung und Fragebogenentwicklung

Ein maßgeschneiderter Online-Fragebogen (Zusatzmaterial online) wurde als Umfrageinstrument verwendet. Die Datenerhebung erfolgte mithilfe des Online-Fragebogen-Tools SurveyMonkey (Fa. SurveyMonkey Inc., San Mateo, CA, USA). Vor seiner Verbreitung wurde der Fragebogen von Notärztinnen und Notärzten der Zentren der Autoren pilotiert, überprüft und validiert, um seine Zuverlässigkeit und Genauigkeit sicherzustellen.

### Fragebogenaufbau

Nach der Erfassung der demografischen Daten und beruflichen Informationen, wie Qualifikation, medizinische Fachrichtung, Institution oder Berufsjahre, wurden die Teilnehmenden gebeten, ihre Meinung zur Notwendigkeit einer Optimierung der aktuellen präklinischen Traumaversorgung abzugeben. Es wurde erfragt, ob sie das Konzept der „Golden Hour“ kennen und ob sie dieses Konzept als angemessen erachten, wobei eine dichotome Antwortauswahl (ja oder nein) verwendet wurde. Darüber hinaus wurden 4 Items verwendet, um die Einstellung zur Verwendung von Blutprodukten und Gerinnungspräparaten in der präklinischen Traumaversorgung allgemein auf einer 5‑Punkte-Likert-Skala (stark zustimmen bis stark ablehnen) zu bewerten. Abschließend wurden die Teilnehmenden gebeten, die Eignung von 9 spezifischen Gerinnungs- und Blutprodukten auf einer 5‑Punkte-Likert-Skala (stark zustimmen bis stark ablehnen) für ihren Einsatz in der präklinischen Traumaversorgung zur Erhöhung der Überlebenswahrscheinlichkeit von Patienten mit Blutverlust zu bewerten.

### Statistik

Da der Fragebogen kategoriale Variablen (nominale oder ordinale) erzeugte, wurden Häufigkeiten und Prozentsätze separat für die beiden Berufsgruppen für jedes Item mittels Kreuztabelle berechnet. Die Statistiksoftware SPSS (IBM Statistics SPSS 29.0, IBM, Armonk, NY, USA) wurde zur Datenverwaltung und zur Datenanalyse verwendet. Zur Visualisierung der Daten wurden gestapelte Balkendiagramme mit Microsoft Excel 2020 (Microsoft Corporation, Redmond, WA, USA) erstellt.

## Ergebnisse

### Charakterisierung der Stichprobe

Insgesamt konnten 9837 Interaktionen mit dem Umfrageaufruf auf den Social-Media-Kanälen verzeichnet werden, wovon *n* = 371 Teilnehmende den Fragebogenlink öffneten. Hiervon verweigerten *n* = 11 ihre informierte Zustimmung, *n* = 6 konnten keiner Berufsgruppe zugeordnet werden, und weitere *n* = 67 Teilnehmende brachen den Fragebogen bei der Erfassung der demografischen Daten vorzeitig ab. Darüber hinaus wurden *n* = 22 Personen wegen fehlender Daten ausgeschlossen. Zusätzlich wurden *n* = 13 Teilnehmende aus der Auswertung, die nicht in Deutschland arbeiteten, ausgeschlossen. Somit bestand die endgültige Stichprobe von den initial 371 eingegangenen Antworten aus *n* = 252 Teilnehmenden, was einem Anteil von 67,9 %, entspricht. Hiervon waren *n* = 110 (43,7 %) Ärztinnen und Ärzte, und *n* = 142 (56,3 %) gehörten dem Rettungsdienstpersonal an. Die Zusammensetzung der Studiengruppe nach Qualifikation ist in Tab. 1 zu finden.

**Tab. 1:** Zusammensetzung der Stichprobe nach Qualifikationen. Aufschlüsselung nach Qualifikation der Teilnehmenden

		Häufigkeit	Prozent (%)
*Ärztinnen und Ärzte*		*n* *=* *110*	*43,7*
*Qualifikation*	Assistenzarzt	22	20
	Facharzt	*49*	44,5
	Oberarzt	33	30
	Medizinischer Direktor	3	2,7
	Sonstige	3	2,7
*Fachrichtung*	Allgemeinmedizin	2	1,8
	Anästhesiologie	91	82,7
	Chirurgie	9	8,2
	Innere Medizin	4	3,6
	Sonstige	4	3,6
*Einrichtung*	Privatpraxis	7	6,4
	Klinik der Grundversorgung	4	3,4
	Klinik der Sekundärversorgung	31	28,2
	Klinik der Maximalversorgung	21	19,1
	Universitätsklinikum	37	33,4
	Sonstige	10	9,1
*Zusatzqualifikation Notfallmedizin*	Ja	100	90,9
	Nein	10	9,1
*Rettungsdienstpersonal*		*n* *=* *142*	*56,3*
*Qualifikation*	Rettungssanitäter	26	18,3
	Rettungsassistent	3	2,7
	Notfallsanitäter	104	73,2
	Sonstige	9	6,3

Der Fragebogen erreichte Teilnehmende aus ganz Deutschland, wobei 50,8 % in Baden-Württemberg arbeiteten. Die durchschnittliche Berufserfahrung betrug *m* = 12,63 (± 7,47) Jahre bei den Ärztinnen und Ärzten sowie *m* = 10,43 (± 8,26) Jahre beim Rettungsdienstpersonal. Von den Ärztinnen und Ärzten verfügten 90,9 % über die Zusatzweiterbildung Notfallmedizin.

### Präklinische Traumaversorgung

Eine große Mehrheit der Ärztinnen und Ärzte (89,1 %) und des Rettungsdienstpersonals (90,8 %) sieht die Notwendigkeit, die präklinische Traumaversorgung zu optimieren. Das Konzept der „Golden Hour“ ist (fast) allen Ärztinnen und Ärzten (100 %) sowie dem Rettungsdienstpersonal (99,3 %) bekannt. Während 80,9 % der Ärztinnen und Ärzte davon ausgehen, dass das Konzept angemessen ist, stimmen rund zwei Drittel des Rettungsdienstpersonals (67,6 %) zu.

### Verwendung von Blutprodukten in der präklinischen Traumaversorgung

**Abb. 1:**
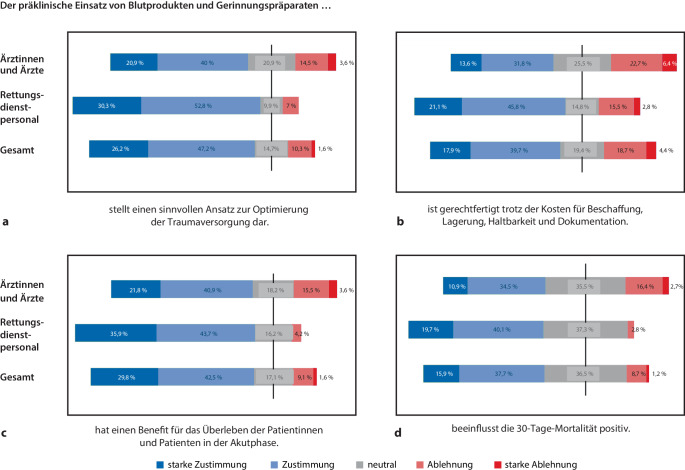
Meinung zur präklinischen Verwendung von Blutprodukten und Gerinnungspräparaten. Antworten der Teilnehmenden auf die Frage nach ihrer Meinung zum allgemeinen präklinischen Einsatz von Blutprodukten und Gerinnungspräparaten im Hinblick auf ihre Eignung zur Optimierung der Traumaversorgung (**a**), ihre Berechtigung trotz verschiedener Kosten (**b**), ihren Nutzen für das Überleben von Patientinnen und Patienten in der Akutphase (**c**) und ihren Einfluss auf die 30-Tage-Mortalität (**d**). Die Daten werden als Prozentsatz der Gesamtantworten (*n* = 252) sowie für Rettungsdienstpersonal (*n* = 142) und Ärztinnen und Ärzte (*n* = 110) dargestellt. Neutrale Antworten sind gleichmäßig durch die *y‑Achse* geteilt, welche 0 % markiert

Unter den Ärztinnen und Ärzten halten 60,9 % den präklinischen Einsatz von Blutprodukten und Gerinnungspräparaten für einen sinnvollen Ansatz zur Optimierung der Traumaversorgung (Zustimmung oder starke Zustimmung), ebenso wie 83,1 % des Rettungsdienstpersonals. Darüber hinaus stimmen 45,4 % der Ärztinnen und Ärzte sowie 66,9 % des Rettungsdienstpersonals zu, dass der präklinische Einsatz dieser Produkte trotz der Kosten für Beschaffung, Lagerung, Haltbarkeit und Dokumentation gerechtfertigt ist. Hinsichtlich des Nutzens in der Akutphase für das Überleben der Patientinnen und Patienten stimmen 62,7 % der Ärztinnen und Ärzte sowie 79,6 % des Rettungsdienstpersonals zu, und weitere 45,4 % der Ärztinnen und Ärzte sowie 59,8 % des Rettungsdienstpersonals gehen davon aus, dass der präklinische Einsatz von Blutprodukten und Gerinnungspräparaten einen positiven Einfluss auf die 30-Tage-Mortalität von Patientinnen und Patienten hat (Abb. 1).

### Eignung von spezifischen Blutprodukten

**Abb. 2:**
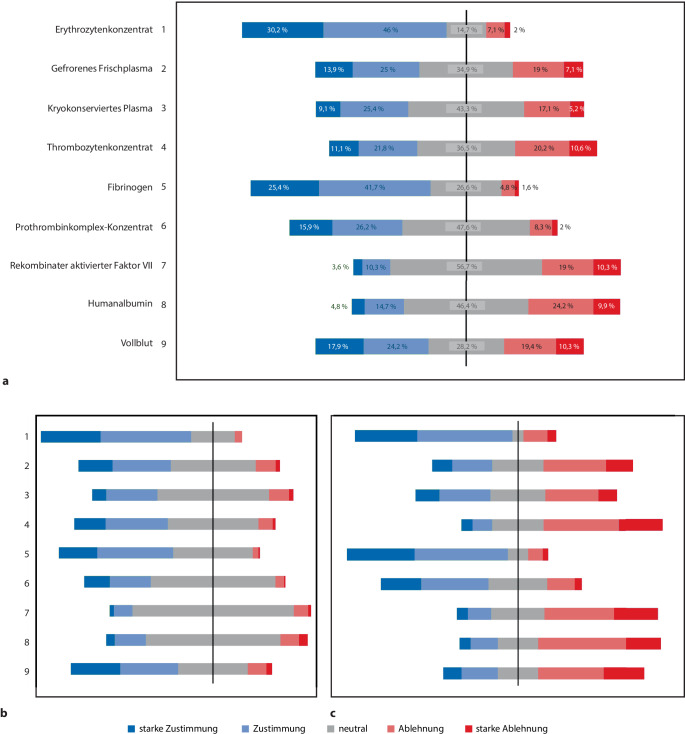
Bewertung von Blutprodukten und Gerinnungspräparaten. Antworten der Teilnehmenden auf die Frage, wie sie die Blutprodukte und Gerinnungspräparate hinsichtlich ihrer Eignung für die präklinische Anwendung bei Traumapatientinnen und -patienten mit Blutverlust bewerten, um die Überlebenswahrscheinlichkeit zu erhöhen. Die Daten werden als Prozentsatz der Gesamtantworten (**a**, *n* = 252) sowie für das Rettungsdienstpersonal (**b,** *n* = 142) und Ärztinnen und Ärzte (**c**, *n* = 110) dargestellt. „Neutrale“ Antworten sind gleichmäßig durch die *y‑Achse* geteilt, welche 0 % markiert

Auf die Frage nach ihrer Meinung zur Eignung bestimmter Blutprodukte für die präklinische Traumaversorgung von Patientinnen und Patienten mit Blutverlust zur Erhöhung der Überlebenswahrscheinlichkeit (Abb. 2; Tab. 2) wurden Erythrozytenkonzentrate (78,2 %), Fibrinogen (80 %) und Prothrombinkomplexkonzentrat (PPSB; 53,6 %) von den Ärztinnen und Ärzten als am besten geeignet eingestuft (Zustimmung oder starke Zustimmung), während Thrombozytenkonzentrat (59,1 %), rekombinanter aktivierter Faktor VII (56,3 %), Humanalbumin (60,9 %) und Vollblut (52,7 %) in dieser Gruppe auf einen großen Anteil an Ablehnung stoßen. Unter dem Rettungsdienstpersonal erhalten Erythrozytenkonzentrate (74,7 %), Fibrinogen (57 %) und Vollblut (53,5 %) die höchste Zustimmung für ihre Verwendung in der präklinischen Traumaversorgung. Im Gegensatz dazu gibt es viele „neutrale“ Antworten für Humanalbumin (66,9 %), rekombinanten aktivierten Faktor VII (80,3 %), PPSB (62 %), kryokonserviertes Plasma (55,6 %) und Thrombozytenkonzentrat (45,1 %).

**Tab. 2:** Bewertung von Blutprodukten und Gerinnungspräparaten. Antworten der Teilnehmenden auf die Frage, wie sie die Blutprodukte und Gerinnungspräparte hinsichtlich ihrer Eignung für die präklinische Anwendung bei Traumapatientinnen und -patienten mit Blutverlust bewerten, um die Überlebenswahrscheinlichkeit zu erhöhen. Die Daten werden als Prozentsatz (%) der Gesamtantworten (*n* = 252) (in kursiver Schrift) sowie separat für Ärztinnen und Ärzte (*n* = 110) sowie Rettungsdienstpersonal (*n* = 142) angegeben

	Starke Zustimmung	Zustimmung	Neutral	Ablehnung	Starke Ablehnung
*Erythrozytenkonzentrat*	*30,2*	*46,0*	*14,7*	*7,1*	*2,0*
Ärztinnen und Ärzte	30,9	47,3	5,5	11,8	4,5
Rettungsdienstpersonal	29,6	45,1	21,8	3,5	0,0
*Gefrorenes Frischplasma*	*13,9*	*25,0*	*34,9*	*19,0*	*7,1*
Ärztinnen und Ärzte	10,0	20,0	25,5	30,9	13,6
Rettungsdienstpersonal	16,9	28,9	42,3	9,9	2,1
*Kryokonserviertes Plasma*	*9,1*	*25,4*	*43,3*	*17,1*	*5,2*
Ärztinnen und Ärzte	11,8	25,5	27,3	26,4	9,1
Rettungsdienstpersonal	7,0	25,4	55,6	9,9	2,1
*Thrombozytenkonzentrat*	*11,1*	*21,8*	*36,5*	*20,2*	*10,3*
Ärztinnen und Ärzte	5,5	10,0	25,5	37,3	21,8
Rettungsdienstpersonal	15,5	31,0	45,1	7,0	1,4
*Fibrinogen*	*25,4*	*41,7*	*26,6*	*4,8*	*1,6*
Ärztinnen und Ärzte	33,6	46,4	10,0	7,3	2,7
Rettungsdienstpersonal	19,0	38,0	39,4	2,8	0,7
*PPSB*	*15,9*	*26,2*	*47,6*	*8,3*	*2,0*
Ärztinnen und Ärzte	20,0	33,6	29,1	13,6	3,6
Rettungsdienstpersonal	12,7	20,4	62,0	4,2	0,7
*Rekomb. akt. Faktor VII*	*3,6*	*10,3*	*56,7*	*19,0*	*10,3*
Ärztinnen und Ärzte	5,5	11,8	26,4	34,5	21,8
Rettungsdienstpersonal	2,1	9,2	80,3	7,0	1,4
*Humanalbumin*	*4,8*	*14,7*	*46,4*	*24,2*	*9,9*
Ärztinnen und Ärzte	5,5	13,6	20,0	43,6	17,3
Rettungsdienstpersonal	4,2	15,5	66,9	9,2	4,2
*Vollblut*	*17,9*	*24,2*	*28,2*	*19,4*	*10,3*
Ärztinnen und Ärzte	9,1	18,2	20,0	32,7	20,0
Rettungsdienstpersonal	24,6	28,9	34,5	9,2	2,8

## Diskussion

Ziel dieser Querschnittstudie war es, die Sichtweise vom Rettungsdienstpersonal sowie von Ärztinnen und Ärzten im deutschsprachigen Raum hinsichtlich der Notwendigkeit einer Verbesserung der präklinischen Traumaversorgung durch die frühzeitige Gabe von Blutprodukten und Gerinnungspräparaten zu untersuchen. Dazu wurde eine große Anzahl von Ärztinnen und Ärzten sowie Rettungsdienstpersonal rekrutiert, die zwischen Oktober 2023 und Januar 2024 einen Online-Fragebogen beantworteten.

Die Studie ergab, dass von den insgesamt 252 Teilnehmenden eine Mehrheit der Ärztinnen und Ärzten (89,1 %) sowie des Rettungsdienstpersonals (90,8 %) die Notwendigkeit einer Verbesserung der präklinischen Traumaversorgung sahen. Dies führte zur Etablierung des „Golden Approach of Trauma“, ein Begriff, der eine Abkehr vom traditionellen Konzept der „Golden Hour of Trauma“ bedeuten soll, dem es an solider wissenschaftlicher Evidenz fehlt [6, 8, 12]. Die rechtzeitige und effektive präklinische Versorgung von Patientinnen und Patienten ist von entscheidender Bedeutung, doch sollten wesentliche notfallmedizinische Maßnahmen nicht zugunsten des Zeitfaktors unterlassen werden [13]. Ein Schwerpunkt der Umfrage war die präklinische Verwendung von Blutprodukten und Gerinnungspräparaten, insbesondere im Zusammenhang mit traumabedingter Koagulopathie und der Notwendigkeit einer angemessenen Sauerstoffversorgung bei hämorrhagischem Schock. Eine frühzeitige Transfusion von Blutbestandteilen kann den pathophysiologischen Prozessen eines Traumas entgegenwirken [14–16]. Obwohl 60,9 % der Ärztinnen und Ärzte sowie 83,1 % des Rettungsdienstpersonals den präklinischen Einsatz von Blutprodukten und Gerinnungspräparaten für eine geeignete Therapie halten, bleibt die Umsetzung auf wenige Standorte beschränkt. Die Datenlage ist nach wie vor uneinheitlich, und es wurde noch kein endgültiges präklinisches Substitutionsprotokoll für eine „damage control resuscitation“ erstellt [17]. Es wurde ein Überlebensvorteil in der akuten Phase nach einem Trauma postuliert, wobei die Meinungen über die Auswirkungen auf die 30-Tage-Mortalität zwischen Ärztinnen und Ärzten (45,4 %) und Rettungsdienstpersonal (66,9 %) auseinandergingen. Es hat sich gezeigt, dass die Verabreichung von Plasma im luftgebundenen Rettungsdienst Patientinnen und Patienten mit dem Risiko eines hämorrhagischen Schocks zugutekommt und die 30-Tage-Sterblichkeit in solchen Fällen verringert [11].

Bezüglich der Transfusion von Erythrozytenkonzentraten stimmen die meisten befragten Ärztinnen und Ärzten mit 78,2 % und ähnlich 74,7 % des Rettungsdienstpersonals in Deutschland dieser Maßnahme zu. Dies deckt sich mit Studien, die darauf hinweisen, dass eine frühzeitige präklinische Verabreichung mit einer erhöhten Überlebenswahrscheinlichkeit korrelieren kann, [18], und dass die frühzeitige Verabreichung von Erythrozytenkonzentraten mit einer höheren Überlebenswahrscheinlichkeit bis zur Krankenhauseinweisung verbunden ist [19]. Ebenso war die präklinische Verabreichung von Erythrozytenkonzentraten im luftgebundenen Rettungsdienst mit einer erhöhten Wahrscheinlichkeit des 24-h-Überlebens verbunden [20]. Bei näherer Betrachtung lässt die Monotherapie mit Erythrozytenkonzentraten die Acidose und die traumainduzierte Koagulopathie ungelöst [21]. Eine präklinische Erythrozytentransfusion kann jedoch effektiv eine Dilutionskoagulopathie, die häufig durch eine übermäßige Verabreichung von kristalloiden Lösungen entsteht, verhindern. Maegele et al. zeigten in einer großen Analyse des deutschen Traumaregisters mit 8724 Patientinnen und Patienten, dass eine Erhöhung des kristalloiden Infusionsvolumens mit einem steigenden Patientenanteil, die eine Koagulopathie aufgrund von eben dieser Dilution entwickeln, verbunden ist [22, 23]. Darüber hinaus verschlimmert die gängige Praxis der Infusion, die zu einer kristalloiden Hämodilution führt, die Hyperfibrinolyse [24]. In der PREDICT-Studie wurde hervorgehoben, dass Gerinnungsstörungen, insbesondere die Hyperfibrinolyse, bereits am Unfallort beginnen und zu erheblichen Veränderungen des pH-Werts, des Lactatspiegels und des Basenüberschusses führen [25]. Bedingt durch Hypoperfusion und/oder Hypoxie wird die Freisetzung von tPA aus Weibel-Palade-Körperchen des Endothels stimuliert und die Hyperfibrinolyse aggraviert [26].

In der vorliegenden Umfrage sprachen sich sowohl Ärztinnen und Ärzte (80 %) als auch das Rettungsdienstpersonal (57 %) für die Verabreichung von Fibrinogen aus. Ziegler et al. zeigten hier in der FInTIC Studie, dass die frühzeitige Fibrinogengabe die Bildung von Blutgerinnseln fördert und dem traumabedingten Fibrinogenabbau entgegenwirkt [27]. Es sind sicherlich weitere Studien erforderlich, um einen möglichen direkten Zusammenhang von frühzeitiger Fibrinogengabe und folglich einer Reduktion von weiteren Transfusionen aufzuzeigen. Ebenso hinsichtlich des Zusammenhangs mit Mortalität und Morbidität sollten hier weiter Studien folgen.

Transfusionsprotokolle im Krankenhaus profitieren von einer effektiven Überwachung der Interventionen mittels Thrombelastographie (TEG) oder Rotationsthrombelastometrie (ROTEM), die sich an zielgerichteten Behandlungsstrategien orientieren [28, 29]. Auch wenn die präklinische Umsetzung dieser Technologien ehrgeizig erscheinen mag, könnte gerade in Hinblick auf die schockinduzierte Endotheliopathie, getriggert durch eine gesteigerte Katecholaminfreisetzung, eine standardisierte hämostatische Diagnostik frühzeitig eine erfolgreiche Therapie ableiten [30]. Diese Endotheliopathie, die als systemische Antwort auf eine traumabedingte Hypoperfusion zu verstehen ist, setzt innerhalb von Minuten ein. Charakteristisch sind hierbei der Verlust der endothelialen Barrierefunktion, Leukozytenadhäsion und Organdysfunktionen [31]. Eine qualitativ hochwertige Infusionsstrategie im Sinne der „damage control resuscitation“ ist unerlässlich. Holcomb et al. wiesen nach, dass mithilfe von Thrombozyten, Plasma und Erythrozytenkonzentraten eine frühzeitige Wiederherstellung der Funktionalität und Hämostase erreicht werden kann [32]. Die Ausweitung der präklinischen Versorgung auf Transfusionen und die Substitution von Blutgerinnungsprodukten stellen jedoch eine logistische Herausforderung dar, die meisten Rettungskräfte halten diesen Aufwand für vertretbar. Jedoch sind 54,6 % der Ärztinnen und Ärzte nicht überzeugt, dass die Kosten gerechtfertigt seien. Speziell die Lagerung von Blutprodukten bedarf einer konsequenten Kühlkette, die vorzugsweise auch mobil vorgehalten werden sollte. Hier ist sicherlich die Skepsis bezüglich der Kosten nachvollziehbar. Natürlich kostet es Arbeitsaufwand und Zeit, ein Transfusionssystem präklinisch zu etablieren. Die Dokumentation per se bindet Arbeitskräfte. Bei Standardisierung eines präklinischen Transfusionssystems kann hier perspektivisch der gesamte Prozess optimiert und konsekutiv können Kosten reduziert werden.

Die Studie zeigte auch eine starke Unterstützung für Erythrozytenkonzentrate (78,2 %), Fibrinogen (67,1 %) und Prothrombinkomplexkonzentrat (PPSB; 42,1 %) als therapeutische Optionen zur Erhöhung der Überlebenschancen. Im Zusammenhang mit traumabedingten Blutungen fanden Zeeshan et al. heraus, dass die Verabreichung von gefrorenem Frischplasma (FFP) in Kombination mit PPSB das Überleben verbesserte und den Transfusionsbedarf, im Vergleich zu FFP allein, verringerte [33]. Goldstein et al. zeigten, dass Patientinnen und Patienten, die Vitamin-K-Antagonisten einnehmen, signifikante Vorteile durch die Gabe von PPSB gegenüber FFP bei dringenden chirurgischen Eingriffen zur Normalisierung der Gerinnung haben [34]. Bouzat et al. fanden in der doppelblinden, randomisierten, placebokontrollierten PROCOAG-Studie keinen statistisch signifikanten Benefit hinsichtlich des 24-h-Blutprodukteverbrauchs nach frühzeitiger Gabe von PPSB, jedoch eine deutlich erhöhte Rate an Thromboembolien. In der PPSB-Gruppe zeigten 56 Patienten (35 %) vs. 37 Patienten (24 %) in der Placebogruppe mindestens ein thrombembolisches Ereignis (absolute Differenz, 11 % [95 %-KI, 1–21 %]; relatives Risiko, 1,48 [95 %-KI, 1,04–2,10]; *P* = 0,03). Jedoch ist anzumerken, dass die PPSB-Gruppe deutlich weniger TXA erhielt (76 % vs. 86 %), ein spezifisches Produkt verwendet wurde (Kanokad®; Laboratoire Français du Biomédicament) und die Studie einem gewissen „survivorship bias“ unterliegt [35].

Bezüglich der prähospitalen Gabe von Plasma sind hier sicherlich zwei randomisierte kontrollierte Studien zu nennen. „Control of Major Bleeding after Trauma (COMBAT)“ und die „Prehospital Air Medical Plasma (PAMPer)“ [11, 36]. In der PAMPer-Studie zeigte sich eine Reduktion der 30-Tages-Mortalität nach Gabe von 2 Einheiten Blutplasma im Vergleich zur Standardversorgung (23 % vs. 33 %, 95-%-Konfidenzintervall [KI]: [−18,6; −1,0], *p* = 0,03). In der COMBAT-Studie zeigte sich kein Unterschied nach 28 Tagen. Bei COMBAT wurden aber nur 32 % der Patienten prähospital mit 2 Einheiten Plasma therapiert, bei PAMPer waren es 89 %. Gemäß dem transfundierten Plasmavolumen betrug der Faktorenanstieg bestenfalls 7 %. „Eine Metaanalyse beider Studien (*n* = 626) zeigte für die Behandlung mit 2 Einheiten Blutplasma eine niedrigere Mortalität nach 24 h (relatives Risiko [RR] 0,69; 95-%-KI: [0,48; 0,99]) bei vergleichbarer Mortalität nach einem Monat (RR 0,86; 95-%-KI: [0,68; 1,11])“ [37].

Es bleibt unklar, wie innerklinische Behandlungskonzepte, wie z. B. die Gabe von Humanalbumin, an präklinische Situationen angepasst werden können, insbesondere bei traumabedingter Koagulopathie (TIC). Nur ein kleiner Teil der Ärztinnen und Ärzte (17,9 %) sowie des Rettungspersonals (21 %) befürwortet den präklinischen Einsatz von Albumin- dies spiegelt die geringe Evidenz und das Fehlen definitiver Studien wider. Es wird vermutet, dass Albumin zur Aufrechterhaltung des intravaskulären Volumens beiträgt, indem es den kolloidosmotischen Druck (COP) erhöht und die Pufferkapazität des Blutes unterstützt. Ob es die präklinische Überlebenswahrscheinlichkeit verbessert, bleibt unklar und muss noch bewiesen werden.

Rekombinanter aktivierter Faktor VII wird bereits klinisch bei schweren unkontrollierbaren Blutungen eingesetzt. Seine therapeutische Wirksamkeit ist verbunden mit thrombembolischen Risiken, die eine sorgfältige Risikobewertung erfordern, zumal hohe Dosen das Risiko arterieller Thromboembolien bei älteren Menschen deutlich erhöhen [38]. Grounds et al. fanden heraus, dass es den Blutverlust deutlich reduziert und die Sterblichkeit bei hämorrhagischen Patientinnen und Patienten senkt [39]. Der ideale Zeitpunkt für den Einsatz ist jedoch noch nicht festgelegt, und der präklinische Einsatz stellt einen „off-label use“ dar.

Vollbluttransfusionen, häufiger im militärischen als im zivilen Bereich [10], werden von 54 % des Rettungsdienstpersonals, aber nur von 28,2 % der Ärztinnen und Ärzte für den präklinischen Einsatz befürwortet. Befürworter argumentieren, dass es sich um eine einfachere Therapieoption als die Blutkomponententherapie handelt. So stellten Murdock et al. fest, dass Vollblut der rekonstituierten Blutkomponententherapie, die mit schlechteren Ergebnissen verbunden sind, überlegen ist [40]. Braverman et al. konnten nachweisen, dass 0+-Vollblut mit niedrigem Titer (LTOWB) zu einer stärkeren Verbesserung des Schockindex (SI) führte und darüber hinaus die Frühsterblichkeit verringerte [41]. Jedoch stellten Avery et al. fest, dass es keinen definitiven Vorteil gegenüber der Komponententherapie gibt [42]. Dass nur ein geringer Teil der Ärzteschaft die Vollbluttransfusion präklinisch befürwortet, mag an der mangelnden Schulung und Praxis in diesem Bereich liegen. Eine bestimmte Patientenklientel, vornehmlich bis dato im militärischen Bereich, in dem nicht mit der zivilen Logistik gearbeitet werden kann, profitiert von der Vollblutgabe. Hier gibt es schon etablierte „Blood-far-forward“-Programme [43, 44]. Im zivilen Kontext spielen unterschiedliche Faktoren eine Rolle – städtische vs. ländliche Struktur, konsekutiv die Dauer bis zum Erreichen eines geeigneten Hospitals und die Anzahl der „echten“ massiven Blutungen/Jahr unterscheiden die einzelnen Rettungsstandorte erheblich. Die genaue Patientenklientel muss hier noch definiert werden.

Diese Ergebnisse unterstreichen die Notwendigkeit, die Bereitschaft des medizinischen Notfallpersonals zur präklinischen Verabreichung geeigneter Transfusions- und Gerinnungsprodukte weiter zu untersuchen. Die bisherige Datenlage zeigt auf, dass nur ein geringer Teil der Patienten von einer präklinischen Transfusion profitieren würde. So zeigten Maegele et al. anhand Daten des TraumaRegister DGU®, dass sich für Deutschland ein jährlicher Bedarf an Blutprodukten für ungefähr 300–1800 Patienten, die von einer prähospitalen Gabe profitieren könnten, ergibt [37]. Ebenso in den USA ergibt die Analyse einen einstelligen Prozentsatz an Patienten, die hiervon profitieren würden [45]. Die laufende wissenschaftliche Debatte konzentriert sich nicht so sehr auf die Frage, ob diese Komponenten verwendet werden sollten, sondern wie dies am besten umgesetzt werden kann, und fordert Diskussionen über geeignete Protokolle oder individuelle Ansätze [46]. Dieser Wandel könnte zur Einführung einer standardisierten Praxis führen, die als „Golden Approach of Trauma“ bezeichnet wird.

## Limitationen

Die Studie weist potenzielle Einschränkungen auf, u. a. hinsichtlich der Auswahl, auch die Verwendung eines nichtstandardisierten Fragebogens kann die Vergleichbarkeit zwischen den Gruppen einschränken und die Zuverlässigkeit beeinträchtigen.

## Schlussfolgerungen

Die Studie zeigt, dass sowohl Ärztinnen und Ärzte als auch Rettungsdienstpersonal die Notwendigkeit sehen, die präklinische Traumaversorgung zu optimieren. Es ist von entscheidender Bedeutung, die Fähigkeiten des Rettungsteams zu nutzen und alle möglichen therapeutischen Optionen einzusetzen, um das Patienten-Outcome zu verbessern. Der Schwerpunkt lag insbesondere auf der präklinischen Verabreichung von Blutprodukten und Gerinnungspräparaten, einschließlich Erythrozytenkonzentrat (RBC), Prothrombinkomplexkonzentrat (PPSB) und gefrorenem Frischplasma (FFP), die weiterführend untersucht werden sollten. In Anbetracht der verfügbaren Daten sollte sich die Debatte von der Frage, ob bestimmte Produkte verwendet werden sollten, verabschieden und auf die Frage verlagern, wie sie im Rahmen eines standardisierten Transfusionsschemas wirksam eingesetzt werden können, um die Überlebenswahrscheinlichkeiten zu verbessern. Es besteht eine klare Bereitschaft, diese Behandlungen einzusetzen; jetzt ist es an der Zeit, diese Optionen präklinisch zu implementieren und den „Golden Approach of Trauma“ zu entwickeln.

## Fazit für die Praxis

- *Kernaussagen*
  Die Mehrheit der Ärztinnen und Ärzte sowie des Rettungsdienstpersonals sieht die Notwendigkeit, die präklinische Traumaversorgung durch die frühzeitige Gabe von Blutprodukten und Gerinnungspräparaten zu verbessern.
  Der „Golden Approach of Trauma“ stellt eine notwendige Weiterentwicklung dar, um die präklinische Versorgung von Traumapatienten effektiver zu gestalten.
  Eine frühe Transfusion von Blutbestandteilen kann die pathophysiologischen Prozesse eines Traumas günstig beeinflussen.
- *Handlungsempfehlungen für lebensbedrohlich blutende Traumata mit (drohendem) Schock*
  Implementierung der präklinischen Transfusion von Erythrozytenkonzentraten, um die Überlebenswahrscheinlichkeit bis zur Klinikankunft zu erhöhen.
  Nach Gabe von Tranexamsäure frühzeitige Verabreichung von Fibrinogen zur Förderung der Blutgerinnselbildung.

## Supplementary Information

ESM Fragebogen

## Data Availability

Daten und Materialien können auf schriftliche Anfrage beim korrespondierenden Autor angefordert werden.

## Abkürzungen

ABCDE: „Airway“, „breathing“, „circulation“, „disability“, „exposure/environment“
COP: Kolloidosmotischer Druck
FFP: Gefrorenes Frischplasma
LTOWB: Niedriger Titer 0+ Vollblut
PPSB: Prothrombinkomplexkonzentrat
RBC: Rote-Blutkörperchen-Konzentrat
ROTEM: Rotationsthrombelastometrie
SI: Schock-Index
TEG: Thrombelastographie
TIC: Traumainduzierte Koagulopathie
tPA: „tissue-type plasminogen activator“
