# Supplementary material for: The golden approach to trauma. Which blood products are needed for optimization of prehospital trauma care?
Source: Anaesthesiologie. 2024 Nov 18;73(12):819–28. [Article in German] doi: 10.1007/s00101-024-01482-6 (PMC11614957; doi:10.1007/s00101-024-01482-6)
Supplement: Supplementary file 1 — ESM Fragebogen [file 101_2024_1482_MOESM1_ESM.pdf]

Zusatzmaterial zum Artikel "The Golden Approach of Trauma. Welche Blutprodukte werden zu Optimierung der präklinischen Traumaversorgung benötigt?" von Bamberg ML, Grasshoff C, Gerstner J et al. 2024 in *Die Anaesthesiologie.*

Zusatzmaterial und Artikel stehen Ihnen auf www.springermedizin.de zur Verfügung. Bitte suchen Sie dort über den Titel.

Golden Approach of Trauma und Prehospital Transfusion

Universitätsklinik für Anästhesiologie und Intensivmedizin Tübingen (UKT)
Notfallmedizinisches Trainingszentrum in Singen (NOTIS e.V.)
Berufsgenossenschaftliche Unfallklinik Tübingen, Klinik für Anästhesiologie (BGU)

* 1. **Sehr geehrte ärztliche Kolleginnen und Kollegen, sehr geehrte Kolleginnen und Kollegen des Rettungsdiensts,**

wir möchten Sie herzlich dazu einladen, an unserer Studie zur Präklinischen Traumaversorgung teilzunehmen.

Im Fokus steht die Therapieoption der präklinischen Transfusion. Mit Ihrer Teilnahme haben Sie die Möglichkeit, wichtige Erkenntnisse zu dieser Behandlungsoption beizutragen und potenzielle Optimierungen in der präklinischen Traumaversorgung zu identifizieren.

Ihre Mitarbeit ist freiwillig und nimmt nur etwa 5 Minuten in Anspruch.

Ihre Antworten werden anonym erfasst und ausgewertet, sodass keine Rückschlüsse auf Ihre Person möglich sind. Ihre Meinung ist uns äußerst wichtig und kann einen wertvollen Beitrag zur Weiterentwicklung der Notfallmedizin leisten.

Die Teilnahme erfordert keine separate Einwilligungserklärung. Wir haben die Anonymität und den Schutz Ihrer Daten sichergestellt.

Sollten Sie Fragen haben, stehen wir Ihnen gerne unter Notfallmedizin@med.uni-tuebingen.de zur Verfügung.

Wir danken Ihnen herzlich im Voraus für Ihre Teilnahme und Ihr Interesse an dieser wichtigen Studie!

Gerne können Sie uns bei Fragen und Anmerkungen jederzeit unter folgender E-Mail Adresse kontaktieren:
notfallmedizin@med.uni-tuebingen.de

Mit freundlichen Grüßen,
Das Studienteam

Universitätsklinik für Anästhesiologie und Intensivmedizin Tübingen (UKT)
Notfallmedizinisches Trainingszentrum in Singen (NOTIS e.V.)
Berufsgenossenschaftliche Unfallklinik Tübingen, Klinik für Anästhesiologie (BGU).

◯ Ich stimme der Verarbeitung und Veröffentlichung der erfassten Daten zu.

◯ Ich stimme der Verarbeitung und Veröffentlichung der erfassten NICHT zu.

Golden Approach of Trauma und Prehospital Transfusion

Demografie

Auswahl ärztliche oder rettungsdienstliche Mitarbeitende

* 2. Ich bin:

◯ Arzt / Ärztin

◯ Rettungsdienstmitarbeiter / in

◯ Weder noch

Golden Approach of Trauma und Prehospital Transfusion

Demografie Arzt/ Ärztin

Wir bitten Sie in diesem Abschnitt allgemeine Angaben zu Ihrer Person zu machen

* 3. In welcher Position arbeiten Sie? Geben Sie hier bitte Ihre höchste Qualifikation/ Funktion an

- ◯ Assistenzarzt / Assistenzärztin
- ◯ Facharzt / Fachärztin
- ◯ Oberarzt / Oberärztin
- ◯ Chefarzt / Chefärztin
- ◯ Sonstiges (bitte angeben)

* 4. Welcher Fachrichtung gehören Sie an?

- ◯ Allgemeinmedizin
- ◯ Anästhesiologie
- ◯ Chirurgie
- ◯ Innere Medizin
- ◯ Sonstiges (bitte angeben)

* 5. In welcher Versorgungseinrichtung sind Sie vor allem beschäftigt?

- ◯ Praxis / Niederlassung
- ◯ Klinik der Grundversorgung
- ◯ Klinik der Regelversorgung
- ◯ Klinik der Schwerpunktversorgung
- ◯ Klinik der Maximalversorgung
- ◯ Universitätsklinikum
- ◯ Sonstiges (bitte angeben)

* 6. Wie viele Jahre Berufserfahrung als Arzt / Ärztin haben Sie?

* 7. Führen Sie die Zusatzbezeichnung Notfallmedizin?

- ◯ Ja
- ◯ Nein

* 8. Seit wie vielen Jahren führen Sie die Zusatzbezeichnung Notfallmedizin?

Golden Approach of Trauma und Prehospital Transfusion

Demografie Rettungsdienstmitarbeiter/in

Wir bitten Sie in diesem Abschnitt allgemeine Angaben zu Ihrer Person zu machen

* 9. In welcher Position arbeiten Sie? Geben Sie hier bitte Ihre höchste Qualifikation/ Funktion an

- ◯ Rettungshelfer/in
- ◯ Rettungssanitäter/in
- ◯ Rettungsassistent/in
- ◯ Notfallsanitäter/in
- ◯ Sonstiges (bitte angeben)

* 10. Wie viele Jahre Berufserfahrung als Rettungsdienstmitarbeiter/in haben Sie?

Golden Approach of Trauma und Prehospital Transfusion

Bundesland

Tätigkeit

* 11. In welchem Bundesland sind Sie tätig?

- ◯ Baden-Württemberg
- ◯ Bayern
- ◯ Berlin
- ◯ Brandenburg
- ◯ Bremen
- ◯ Hamburg
- ◯ Hessen
- ◯ Mecklenburg-Vorpommern
- ◯ Niedersachsen
- ◯ Nordrhein-Westfalen
- ◯ Rheinland-Pfalz
- ◯ Saarland
- ◯ Sachsen
- ◯ Sachsen-Anhalt
- ◯ Schleswig-Holstein
- ◯ Thüringen
- ◯ Sonstiges (bitte angeben)

Golden Approach of Trauma und Prehospital Transfusion

Traumaversorgung allgemein

Bitte beantworten Sie nun einige allgemeine Fragen zur Versorgung von Traumapatienten.

* 12. Sehen Sie Bedarf zur Optimierung der präklinischen Traumaversorgung?

- ◯ JA
- ◯ NEIN

* 13. Kennen Sie den Begriff "Golden Hour" in der präklinischen Traumaversorgung?

- ◯ JA
- ◯ NEIN

* 14. Halten Sie das Konzept der "Golden Hour" der präklinischen Traumaversorgung für zeitgemäß?

- ◯ JA
- ◯ NEIN

Golden Approach of Trauma und Prehospital Transfusion

Präklinische Blut- und Gerinnungsprodukte

Bitte beantworten Sie nun einge Fragen zur Verwendung von Blut-und Gerinnungspräperaten.

* 15. Bewerten Sie die folgenden Aussagen zur Präklinischen Verwendung von Blutprodukten und Gerinnungspräparaten:

	Starke Zustimmung	Zustimmung	Neutral	Ablehnung	Starke Ablehnung
Die Möglichkeit zur präklinischen Verwendung von Blutprodukten und Gerinnungspräperaten stellt einen sinnvollen Ansatz der Optimierung der Traumaversorgung dar.	◯	◯	◯	◯	◯
Ich halte einen präklinischen Einsatz von Blutprodukten und Gerinnungspräperaten trotz Beschaffungs-, Lagerungserfordernissen und Überprüfung von Haltbarkeit und dem damit verbundenen Dokumentationsaufwand für gerechtfertigt.	◯	◯	◯	◯	◯
Ich sehe einen Benefit in der präklinische Transfusion von Blutprodukten und Gerinnungspräperaten in der Akutphase für das Überleben des Patienten.	◯	◯	◯	◯	◯
Ich bin der Meinung, dass die Gabe von Blut-produkten und Gerinnungs- präperaten in der präklinischen Versorgung die 30-Tage-Mortalität positiv beeinflusst.	◯	◯	◯	◯	◯

* 16. Die folgenden Blutprodukte und Gerinnungspräperate sind für den präklinischen Einsatz bei Traumapatienten mit Blutverlust geeignet um die Überlebenswahrscheinlichkeit zu erhöhen:

	Starke Zustimmung	Zustimmung	Neutral	Ablehnung	Starke Ablehnung
Erythrozyten-konzentrat (LAE)	○	○	○	○	○
Fresh Frozen Plasma	○	○	○	○	○
Plasma (kryo-konserviert)	○	○	○	○	○
Thrombozytenkonzentrat	○	○	○	○	○
Fibrinogen	○	○	○	○	○
Prothrombinkomplex-Konzentrat (PPSB)	○	○	○	○	○
Rekombinanter aktivierter Faktor VII (NovoSeven)	○	○	○	○	○
Humanalbumin	○	○	○	○	○
Vollblut	○	○	○	○	○

17. Vielen Dank für die Teilnahme an der Studie! Falls Sie Anmerkungen dazu haben, können Sie diese gerne nachfolgend hinterlassen:
